# Therapeutic Potential of Porcine Liver Decomposition Product: New Insights and Perspectives for Microglia-Mediated Neuroinflammation in Neurodegenerative Diseases

**DOI:** 10.3390/biomedicines8110446

**Published:** 2020-10-22

**Authors:** Tamotsu Tsukahara, Hisao Haniu, Takeshi Uemura, Yoshikazu Matsuda

**Affiliations:** 1 Department of Pharmacology and Therapeutic Innovation, Nagasaki University Graduate School of Biomedical Sciences, Nagasaki 852-8521, Japan; 2 Institute for Biomedical Sciences, Interdisciplinary Cluster for Cutting Edge Research, Shinshu University, Matsumoto 390-8621, Japan; hhaniu@shinshu-u.ac.jp (H.H.); tuemura@shinshu-u.ac.jp (T.U.); 3 Division of Gene Research, Research Center for Supports to Advanced Science, Shinshu University, Nagano 390-8621, Japan; 4 Division of Clinical Pharmacology and Pharmaceutics, Nihon Pharmaceutical University, Saitama 362-0806, Japan; yomatsuda@nichiyaku.ac.jp

**Keywords:** microglia, porcine liver decomposition product, lysophospholipids, mild cognitive impairment, dementia, neuroinflammation, cytokines, oxygen reactive species

## Abstract

It is widely accepted that microglia-mediated inflammation contributes to the progression of neurodegenerative diseases; however, the precise mechanisms through which these cells contribute remain to be elucidated. Microglia, as the primary immune effector cells of the brain, play key roles in maintaining central nervous system (CNS) homeostasis. Microglia are located throughout the brain and spinal cord and may account for up to 15% of all cells in the brain. Activated microglia express pro-inflammatory cytokines that act on the surrounding brain and spinal cord. Microglia may also play a detrimental effect on nerve cells when they gain a chronic inflammatory function and promote neuropathologies. A key feature of microglia is its rapid morphological change upon activation, characterized by the retraction of numerous fine processes and the gradual acquisition of amoeba-like shapes. These morphological changes are also accompanied by the expression and secretion of inflammatory molecules, including cytokines, chemokines, and lipid mediators that promote systemic inflammation during neurodegeneration. This may be considered a protective response intended to limit further injury and initiate repair processes. We previously reported that porcine liver decomposition product (PLDP) induces a significant increase in the Hasegawa’s Dementia Scale-Revised (HDS-R) score and the Wechsler Memory Scale (WMS) in a randomized, double-blind, placebo-controlled study in healthy humans. In addition, the oral administration of porcine liver decomposition product enhanced visual memory and delayed recall in healthy adults. We believe that PLDP is a functional food that aids cognitive function. In this review, we provide a critical assessment of recent reports of lysophospholipids derived from PLDP, a rich source of phospholipids. We also highlight some recent findings regarding bidirectional interactions between lysophospholipids and microglia and age-related neurodegenerative diseases such as dementia and Alzheimer’s disease.

## 1. Introduction

Amnesic patients with mild cognitive impairment (MCI) are at risk of developing dementia. Approximately 15–20% of people aged 65 or older have MCI [1]. Alzheimer’s disease, Lewy body dementia, and vascular dementia are the most common forms of dementia; both are preceded by a stage of cognitive impairment [2,3]. However, some individuals with amnesia and MCI revert to normal cognition or do not deteriorate further. While clinical studies are currently being conducted to identify novel therapeutics to improve symptoms and prevent or delay the progression of MCI to dementia, no therapeutic drugs have been approved for MCI thus far. Therefore, further studies are needed to determine the causes of MCI and risk factors of progression from MCI to dementia. It has been reported that lipids are increasingly recognized for their roles in neuronal function in the brain [4]. Indeed, the healthy human brain is composed of nearly 60% lipids, which is higher than in any other tissue [5]. Their work also showed that the relative abundance of phosphatidylcholine (PC), phosphatidylserine (PS), and phosphatidylethanolamine (PE) varies between the white and gray matter in the brain [5]. Phospholipids are important components of all mammalian cells; they have a variety of biological functions in the brain and serve as precursors for various secondary messengers such as arachidonic acid (AA), eicosapentaenoic acid (EPA), docosahexaenoic acid (DHA), ceramide, phosphatidic acid (PA), and lysophosphatidic acid (LPA). Lysophospholipid mediators have long been recognized as membrane phospholipid metabolites [6]. They belong primarily to one of six classes based on the structure of the lipid headgroup: LPA, lysophosphatidylcholine (LPC), lysophosphatidylethanolamine (LPE), lysophosphatidylglycerol (LPG), lysophosphatidylinositol (LPI), and lysophosphatidylserine (LPS). Each class has distinct biological functions dependent on physiological adaptation and availability of their respective cell surface receptors [7]. Lysophospholipids have been shown to induce a wide variety of biological effects, including cell proliferation, calcium signaling, metabolic activity, inflammatory and anti-inflammatory processes, and neurite formation [8,9,10,11,12,13]. These effects are generally evoked through receptor–ligand interactions, but this is not always the case. In addition to signaling via receptors, high physiological concentrations of LPC (approximately 150–200 μM in body fluids) suggest that they can alter membrane properties or interact directly with proteins in ways other than saturable binding to a ligand-specific site [13]. Furthermore, lipid head-group specificity in these instances may be due to the net charge of the lipid head groups. This net charge may affect the viscosity of the lipid bilayer or allow interactions with lipolytic enzymes, such as phospholipase A_2_ (PLA_2_) and phospholipase D (PLD), which convert these structural phospholipids into regulatory messengers and subsequently influence neurotransmission [14]. Previous evidence has indicated that cyclic phosphatidic acid (cPA) is generated in mammalian cells by phospholipase D_2_ (PLD_2_) from LPC in vitro and in vivo and mimics the effects of activating signaling pathways similar to those of neurotrophin NGF [15]. In addition, cPA effectively attenuates demyelination, glial activation, and motor dysfunction in an animal model of multiple sclerosis [16]. These studies suggested that phospholipids could be promising therapeutic agents for neurodegenerative diseases. Thus, it is critically important to focus our research efforts on PLDP in older individuals. 

## 2. Microglia in Neurodegeneration 

**Figure 1:**
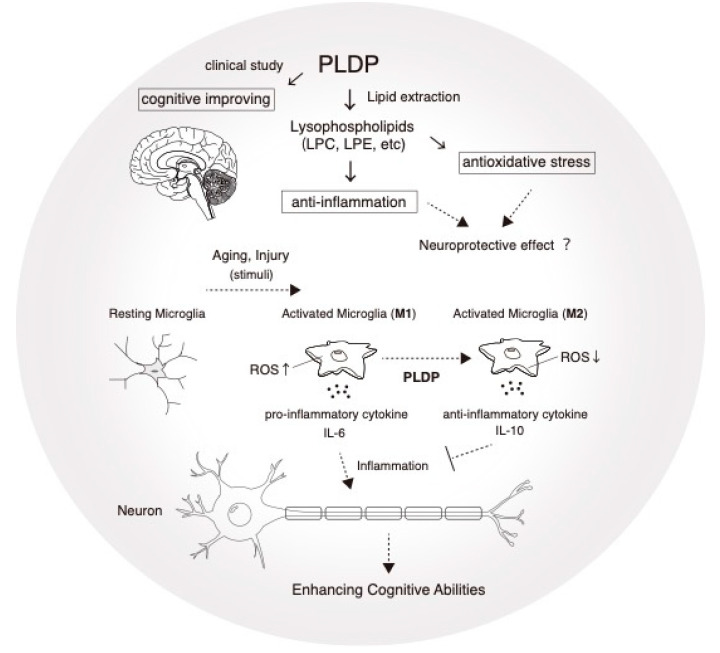
A potential role in the mechanism of action of PLDP in microglial cells. Our previous study suggested that PLDP improve cognitive function at older ages [29], by acting as a rich source of lysophospholipids (LPLs). Total phospholipids were extracted from PLDP using the Bligh and Dyer method, an analysis of the composition of total phospholipids from PLDP revealed that the most abundant LPLs was LPC and LPE. We measured LPS-mediated anti-inflammatoly cytokines expression and ROS production with or without PLDP-derived LPLs treatment, IL-6 expression and ROS production was decreased.Conversion to the activated microglial phenotype (M1 and M2) is often accompanied by the release of NO and reactive oxygen species (ROS), along with the production of inflammatory cytokines such as IL-1, IL-6, and tumor necrosis factors (TNFs). This inflammatory milieu creates a toxic environment that leads to neuronal dysfunction and death. PLDP is a rich source of lysophospholipids including lysophosphatidylcholine (LPC) and lysophosphatidylethanolamine (LPE). In contrast, anti-inflammatory cytokines such as IL-10 and IL-4 induce the M2 phenotype, which possesses neuroprotective properties. We identified novel cooperative actions of lysophospholipids resulting in inhibition of IL-6 secretion and intracellular ROS accumulation in microglia after LPS-induced neuroinflammation. PLDP represents a promising nutraceutical that could improve cognitive function.

Neurodegeneration is an age-dependent progressive deterioration of neuronal components and functions, ultimately leading to cognitive impairment and dementia [17]. These changes occur due to genetic mutations or protein-misfolding diseases such as Alzheimer’s disease that can accumulate with age [18]. Many groups have clearly demonstrated the close spatial–temporal relationship network between amyloid fibrils and activated microglia in both Alzheimer’s disease patients and animal models [19,20,21]. Several studies have also indicated that Alzheimer’s risk genes determine the microglial response to amyloid-β precursor protein but not to Tau pathology using single microglia sequencing [22,23]. These results suggest that microglia are associated with the progression of Alzheimer’s disease. Activation of microglia has been extensively documented as an early event in the pathogenesis of protein-misfolding diseases [24]. These reports suggest that microglia are important aspects of CNS homeostasis and injury repair in aging. Under pathological conditions, such as altered neuronal function, injury, ischemia, and inflammation, microglia become activated, proliferate, and change from a ramified to an amoeboid cell type [25]. Microglial activation has been shown to lead to two opposing cell states, namely classical (M1) and alternative (M2) activation. The M1 phenotype is considered to be a pro-inflammatory state, in which microglial cells produce and release reactive oxygen species (ROS) and cytokines [26]. This is particularly evident in neurodegenerative disorders that involve protein aggregation events such as Alzheimer’s disease. Activated microglia play a potentially detrimental role by eliciting the expression of pro-inflammatory cytokines, such as interleukin 1 beta (IL-1β), IL-6, and tumor necrosis factor-alpha (TNF-α), affecting the surrounding brain tissue [27]. Under ischemic stress, microglia are activated and produce high levels of ROS, which are known to induce oxidative injury in neurovascular cells [28]. In contrast, the M2 microglial phenotype is considered an anti-inflammatory effect and is involved in the production and release of pleiotropic cytokines and neurotrophins, such as IL-10, IL-4, and TGF-β, and low levels of pro-inflammatory cytokines. Anti-inflammatory cytokines such as IL-10 and IL-4 induce the M2 phenotype, which possesses neuroprotective properties. Microglia have become more important than ever in demonstrating strong genetic implications for microglia molecules and the immune system (Figure 1). These studies indicate that understanding human microglial function in neurodegenerative diseases may elucidate new targeted therapies.

## 3. Mitochondrial Dysfunction and Neurodegenerative Disease 

The inflammatory response is crucial in controlling and counteracting the harmful effects triggered by a variety of insults to the central nervous system. However, severe or chronic neuroinflammation can damage the central nervous system (CNS) because of excessive microglial production of cytokines and other inflammatory mediators, such as ROS. More recently, evidence has emerged for impaired mitochondrial dynamics in neurodegenerative diseases such as Alzheimer’s disease. Mitochondria are responsible for ATP generation, ROS formation, intracellular Ca^2+^ homeostasis, and cell death [30]. The mitochondrial cascade hypothesis includes oxidative stress and overproduction of oxidative free radicals such as ROS and reactive nitrogen species [31]. ROS are toxic by-products generated in the mitochondria, and excess ROS may contribute to age-related disease. Impaired mitochondrial function and associated bioenergetic changes alter Alzheimer’s disease homeostasis and lead to an accumulation of amyloid β-protein. Damage to mitochondria leads to a deficiency in energy production, oxidative stress, inflammation, and neuronal damage [32]. Microglia are resident macrophages and play a central role in Alzheimer’s disease-related inflammation. It has been reported that microglial cells internalize aggregates of the Alzheimer’s disease amyloid β-protein via the scavenger receptor CD36 [30]. Microglia have been reported to play a central role as moderators of amyloid β-protein degradation or clearance [33]. Recent evidence indicates that decreased clearance of amyloid β-protein is the driving force leading to its toxic accumulation in Alzheimer’s disease. Amyloid β-protein interacts with microglia via CD36 and a heterodimer of toll-like receptor (TLR) 4 and 6. This interaction seems to activate the NLR family pyrin domain containing 3 (NLRP3) inflammasome, which results in the secretion of IL-1β [34]. The resulting overexpression of IL-1β can aggravate the chronic inflammatory response in the CNS [35]. These findings suggest that NLRP3 and IL-1β play important roles in the pathophysiology of Alzheimer’s disease. 

## 4. PLDP and Cognitive Improvement 

To take advantage of the by-products of the porcine industry, porcine livers are used to obtain protein hydrolyzates. Porcine liver decomposition product (PLDP) is produced by hydrolysis, carried out using commercially available proteases and performed under optimal conditions [29]. After hydrolysis, proteases are heat-inactivated. Following the filtration and washing steps, a filter cake is formed. It has been reported that peptides from porcine liver hydrolyzates have antioxidant and angiotensin-converting enzyme (ACE) inhibitory properties [36]. Reductions in serum cholesterol have been observed following liver phospholipid treatment [37]. These data suggest that the liver has a high nutritional content and offers a good source of protein, lipids, and carbohydrates [38]. Our recent study demonstrated that PLDP can improve cognitive function in older adults by providing a rich source of phospholipids and lysophospholipids [29]. These findings suggest that PLDP is a promising nutraceutical for healthy adults over 40 years of age. We have previously demonstrated that PLDP primarily consists of phospholipids [29]. The components and amount of PLDP administered daily are shown in Table 1. An analysis of the composition of PLDP revealed that the most abundant phospholipids belonged to the PC class. Within this class, PC was the most abundant phospholipid, followed by PE. Other identified phospholipids included PS, PI, and PA. Likewise, extracted ion chromatogram (EIC) analysis of LPCs in the PLDP showed that the most abundant LPC was LPC (18:0). The most abundant LPE was LPE (18:0), while the most abundant LPI detected was LPI (18:0). The most abundant LPS detected was LPS (18:0), and the most abundant LPA detected was LPA (18:0) [39]. PLDP primarily includes phospholipids [29]. Phospholipids are structurally and functionally important cell membrane constituents. Numerous studies have been conducted to examine their role in aging [40], as phospholipids are the primary functional components of neuronal membranes [41]. These results suggest that PLDP represents a promising nutraceutical that could improve cognitive function in healthy adults over 40 years of age. Phospholipids, including PC, PE, PI, PS, and PA, are composed of a diacylglycerol moiety attached to a phosphate group, which in turn is connected to various head groups. Two acyl chains derived from fatty acids are attached to the first and second carbons of the glycerol moiety, denoted as sn-1 and sn-2, respectively [42]. Multiple isoforms of PLA_2_ have been reported to hydrolyze PC, PS, and PE at the sn-2 position to form lysophospholipids, including LPA, LPC, LPS, and LPE [43]. Phospholipids are major constituents in the intestinal lumen after meal consumption and products of phospholipid metabolism in the intestine through PLA2- and ATX-mediated pathways [44]. Oral medication administration is one of the preferred routes for patients. Many drugs can be administered orally as capsules, tablets, or liquids. Since oral administration is the most convenient, safest, and least expensive route, it is most often used. Phospholipids derived from food sources (e.g., PLDP), especially complex lipids, are also capable of affecting gastrointestinal function and the enteric nervous system [45]. In addition, the digestive tract releases multiple types of bioactive lipids into the intestinal lumen [46]. Therefore, gastrointestinal inflammation is affected by both dietary lipids and lipid metabolism in the digestive tract [47]. Modulation of gastrointestinal wound repair and acute inflammation by mucus phospholipids in tissue is well understood [48]. In addition, systemic inflammation can increase the levels of pro-inflammatory cytokines in the CNS associated with glial activation in neurodegeneration [49]. LPC and LPA, which are also produced via PC hydrolysis, increase alpha-7 nicotinic acetylcholine receptor signaling and improve cognitive function by increasing long-term potentiation, increasing synaptic excitability [50]. Lysophospholipids mediate signaling, proliferation, neural activity, and inflammation, thus contributing to the regulation of a variety of important pathophysiological processes, including cerebral ischemia, vascular dementia, and Alzheimer’s disease [51]. Under normal circumstances, inflammation is a protective response that facilitates the healing process [52]. However, leukocytes and macrophages are found throughout the blood and cytokines are secreted by cells of the nervous system as part of an immune-modulating response that helps to resolve inflammation [53]. Neuroinflammation is a hallmark of all major CNS diseases. The main mediators of neuroinflammation are microglial cells, which are activated during CNS injuries (e.g., stroke and spinal cord injury). Microglial cells initiate a rapid response that involves cell migration, proliferation, and release of cytokines, chemokines, and neurotrophic factors [54]. In addition, microglia can release potent neurotoxins that cause neuronal damage [55]. Since mitochondrial dysfunction is involved in neuroinflammation, repair and support of this organelle is critical in biological systems. Mitochondria have a lipid membrane, similar in composition to the plasma membrane, which can be damaged by the production of reactive oxygen species (ROS). Excess free-radical species adversely modify cell components, exacerbating lipid, protein, and DNA damage that underlie multiple pathogenic conditions. Induced lipid peroxidation also plays a critical role in cell death pathways, including apoptosis [56]. Therefore, exogenous phospholipids can provide an important source of bioactive components for the repair of these membranes [57]. We anticipate that a detailed study of the biological activities of phospholipids identified in PLDP will clarify the currently unknown pathophysiological mechanisms underlying dementia.

## 5. Lysophospholipids and Neuroinflammation

Based on the results of research over the last two decades, lysophospholipids have served not only as structural components of biological membranes, but also as biologically active molecules. They are known to influence a broad variety of processes, including neurogenesis, immunity, vascular development, and regulation of metabolic diseases [15,44,58,59,60]. With growing interest in the involvement of extracellular lysophospholipids in both normal physiology and pathology, it has become increasingly evident that these small mediators may have therapeutic potential for anti-neurodegenerative indications. It has been demonstrated that the lipopolysaccharide (LPS)-mediated inflammatory response, including increased microglial cytokine production, is significantly suppressed by LPC or LPE exposure [39,61]. Microglial activation leads to the production of pro-inflammatory cytokines, including IL-1, IL-6, and TNF-α [25]. A recent study suggested that LPC treatment attenuated the increased expression of the pro-inflammatory cytokines IL-1β, IL-6, and TNF-α, which suggests that it may be neuroprotective and/or protect against neuroinflammation. Previous studies have suggested that the pro-inflammatory cytokine TNF-α is a primary mediator of the inflammatory response that stimulates the synthesis and release of other cytokines [62]. Our results showed that the LPS-mediated inflammatory response, including the observed increase in microglial cytokine production, was suppressed by LPC exposure. This finding is important given that microglia-mediated neuroinflammation is regarded as a pathological mechanism in many neurodegenerative diseases and a key event accelerating cognitive or functional decline. While the release of these factors is typically intended to prevent further damage to CNS tissues, they may be toxic to certain neurons and other glial cells [25]. These studies suggest that understanding the role of proinflammatory cytokines in neurodegenerative diseases is complicated by cytokines possessing dual roles in neuroprotection and neurodegeneration. For example, IL-6 plays roles in aging, brain injury, dementia, and autoimmune disease [63]. IL-6 is a pleiotropic cytokine that is often undetectable in the normal brain. Its acute release in response to injury is well studied and documented [64]. LPS usually stimulates IL-6 production in both astrocytes and microglia, while TNF-α induces IL-6 in astrocytes, but not microglia [64]. There may be species-specific effects in which LPS mostly affects TNFα, IL-1β, and IL-6 production in microglia rather than in astrocytes, although IL-1β stimulates IL-6 synthesis in astrocytes [65]. Previous studies have also indicated that the anti-inflammatory cytokine IL-10 was induced by LPC or LPE exposure in ventral mesencephalic neurons and microglia in response to LPS stimulation [66,67]. IL-10 is known to inhibit the LPS-induced production of several inflammatory mediators [68]. Furthermore, it is an important modulator of neuronal homeostasis, with anti-inflammatory and neuroprotective functions, which can be released by activated microglia [69]. Neuroinflammation is a major pathogenic condition that affects the onset and progression of neurodegenerative diseases [70]. Inhibition of inflammatory cytokine production may play a role in the anti-inflammatory activity of lysophospholipids, suggesting novel therapeutic applications.

## 6. Oxidative Stress and Lysophospholipids

Oxidative stress is an important phenomenon caused by an imbalance between the production and degradation of free radicals and ROS in cells, leading to accumulation [71]. ROS are known to damage all cellular macromolecules (lipids, nucleic acids, and proteins). This damage leads to secondary metabolic activities that can be just as damaging as the initial ROS [72]. Oxidative stress seems to be involved in the pathogenesis of both major types of dementia: Alzheimer’s disease and vascular dementia [73]. In Alzheimer’s disease, oxidative modifications are closely associated with subtle inflammatory processes in the brain [74]. The brain becomes extremely enriched in oxidized phospholipids [75]. Lipid peroxidation is one of the major outcomes of free radical-mediated injury in neurological disorders [76]. Polyunsaturated fatty acid peroxidation triggers a devastating sequence of reactions in cell membranes. Once oxidative damage is initiated, ROS oxidize polyunsaturated fatty acids in the cell membrane in a self-propagating chain reaction. The cell membrane, which is composed of polyunsaturated fatty acids, is a primary target for ROS attack, leading to cell membrane damage. Increased ROS production is harmful, leading to adverse oxidative modifications to cell components, such as mitochondrial structures, which are sensitive targets of ROS-induced damage [77]. Polyunsaturated fatty acids can be further modified by cyclooxygenases to form prostaglandins, which are unstable and can be converted into various prostanoids depending on the cellular regulation of terminal prostanoid synthesis pathways [78]. On the other hand, LPC is a type of bioactive lysophospholipid that circulates in the body at high concentrations. LPC and LPE are highly abundant phospholipid components of PLDP that incorporate choline or phosphatidylethanolamine as their head group. PC hydrolysis at the sn-2 position by the superfamily of PLA_2_ enzymes generates LPC [79]. The mechanisms of neuronal death in the disease remain unclear, although it has been postulated that cell death is due to apoptosis [80]. Reports have linked apoptosis to an increase in mitochondrial oxidative stress that causes cytochrome c release, subsequent caspase activation, and cell death [81,82,83]. A previous report suggested that LPC inhibits hydrogen peroxide-induced apoptosis in macrophages [84]. It has also been reported that LPE displays anti-inflammatory action when orally administered in zymosan-induced peritonitis, which is a model commonly used to study systemic inflammatory response syndrome [85]. LPE has been detected in human serum at concentrations of about several hundred nanograms per milliliter [86] and has been reported to function as an intercellular signaling molecule [87]. Numerous studies suggest that besides amyloid β-protein accumulation, dysregulation of intracellular Ca^2+^ homeostasis might act as an important progenitor of Alzheimer’s disease. Reduced serum calcium levels are associated with the conversion of MCI to early Alzheimer’s disease [88]. Many factors and signaling pathways that are activated by inflammation and oxidative stress are involved in the propagation of neurodegenerative diseases [89]. Our previous study suggested that 1-O-alkyl glycerophosphate (AGP), an ether-linked LPA, is present in the brain and spinal cord, and plays a crucial role during nervous system development. Tokumura described a phospholipase D (PLD) responsible for the degradation of lyso derivatives of platelet-activating factor (PAF) to AGP [90]. In addition, we found that AGP treatment increased the production of intracellular ROS and induced peroxisome proliferator-activated receptor γ (PPARγ) activation in microglial cells [91]. PPARγ is a member of the nuclear hormone receptor superfamily, many of which function as ligand-activated transcription factors. Intriguingly, AGP upregulated the expression levels of CD36 class B scavenger receptor, a high-affinity receptor for oxidized low-density lipoproteins (LDL). These findings suggest that AGP induces PPARγ activation, enhances CD36 expression, and increases the production of intracellular ROS in microglial cells. We showed that AGP strongly induced ROS-mediated microglial cell activation. ROS production in microglial cells was significantly increased upon treatment with AGP, and this production was attenuated by a synthetic irreversible PPARγ antagonist. Additionally, PPARγ-induced and CD36-mediated microglial amyloid-β phagocytosis results in cognitive enhancement [91]. It is well known that inflammatory cytokines and signaling pathways play pivotal roles in microglial activation [92]. CD36-mediated ROS generation is an important factor that mediates AGP-induced cytokine signaling in microglial cells. These results indicate that inflammatory processes play a role in neurodegenerative disease progression and pathology, and that an extract of PLDP containing a mixture of physiologically and pharmacologically active phospholipids exerted potent anti-inflammatory and anti-oxidative effects against neurodegeneration underlying dementia. These studies suggest that lysophospholipids are of particular interest for their anti-inflammatory properties.

## 7. Conclusions and Future Perspectives

**Figure 2:**
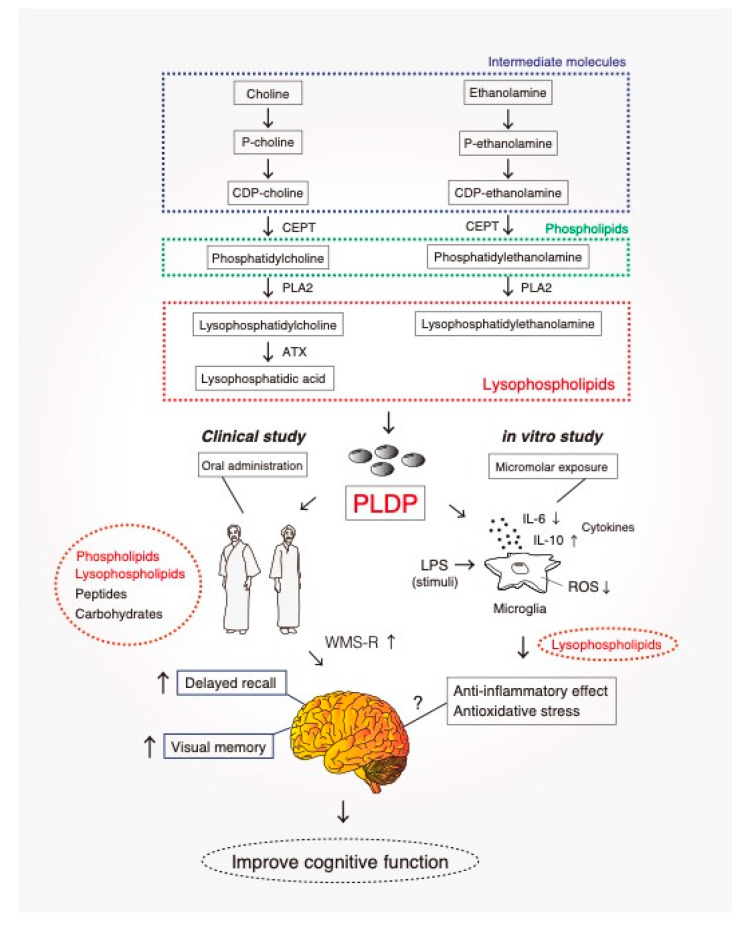
Effect of PLDP in vitro and in vivo. Oral administration of PLDP to healthy study participants (over 40 years of age) enhanced Visual Memory and Delayed Recall. Our primary focus was to identify a functional phospholipid contained within PLDP, confirm its clinical effects, and develop it as a new drug for treating neurodegenerative diseases, including dementia. PLDP: porcine liver decomposition product, CEPT: choline/ethanolamine phosphotransferase, PLA_2_: Phospholipase A_2_, ATX: Autotaxin, also known as ectonucleotide pyrophosphatase/phosphodiesterase family member 2, WMS-R: Wechsler Memory Scale.

Accumulating evidence indicates that dementia is caused by damage to microglia–neuron communication [93]. Once activated, microglia can be potent immune effector cells that initiate both innate and adaptive immune responses and produce a number of cytokines, chemokines, and growth factors [94]. There has been increasing interest in the role of inflammation as a common mechanism of disease, including neurodegeneration. Common neurodegenerative diseases, including dementia, are associated with chronic neuroinflammation. This inflammation and subsequent tissue damage interfere with the ability of brain cells to communicate with each other. Signs and symptoms are linked to three stages of dementia: the early stage, middle stage, and late stage. A strong link has been observed between pro-inflammatory cytokines and neurodegeneration, both in clinical data and basic science research [95]. Recent evidence suggests that midlife risk factors for cardiovascular disease, including high cholesterol, hypertension, high homocysteine, and inflammation, are important risk factors for dementia in later years [96]. Furthermore, high cholesterol and hypertension have consistently displayed an association with increased risk of Alzheimer’s disease and vascular dementia [97]. Vascular pathology plays a key role in the development of vascular dementia in addition to Alzheimer’s disease [98]. An interesting report indicated that LPC and LPS attenuate the expression of inflammatory mediators in atherosclerosis [99]. Unfortunately, no treatment is currently available to cure dementia or to alter its progression. Numerous new treatments under investigation are in clinical trials. Currently, the principal goal of dementia care is early diagnosis, to promote early and optimal management. Although the exact mechanisms of action of PLDP are not fully understood, one possibility is a synergistic effect of phospholipid components in PLDP (Figure 1). Currently, LPC and LPE are the most attractive research targets in terms of biological activity and possible applications. Enzymatic digestion of LPC leads to the formation of various forms of bioactive lipids such as LPA and cPA, which are involved in modulating cardiovascular system physiology, wound healing, and carbohydrate metabolism, mediated by plasma membrane and nuclear receptors [15,100]. LPE is likely to have neuroprotective roles both in vivo and in vitro. We showed that media conditioned via exposure to LPE-treated microglia promoted morphological changes in a dose-dependent manner. Our study also indicated that LPC and LPE synergistically suppressed LPS-stimulated ROS production [39]. This finding is important given that microglia-mediated neuroinflammation is regarded as a pathological mechanism in many neurodegenerative diseases, such as dementia and Alzheimer’s disease, and a key event in accelerating cognitive or functional decline. Thus, lysophospholipids a promising therapeutic candidate for the treatment of age-related cognitive impairments, including neurodegenerative diseases, such as dementia (Figure 2). The effect of PLDP and its component phospholipids on other mental diseases will be an interesting topic to explore. We aimed to identify a functional phospholipid contained within PLDP that has been confirmed to have clinical effects and to develop a new drug for treating dementia and Alzheimer’s disease.

## Figures and Tables

**Table 1:** The components and the amount of PLDP administered daily are shown. PC: phosphatidylcholine, PE: phosphatidylethanolamine, PI: phosphatidylinositol, PS: phosphatidylserine, PA: phosphatidic acid, SM: sphingomyelin, LPC: lysophosphatidylcholine.

Composition		Amount/Day
Phospholipids	PC	16.8 mg
	PE	4.0 mg
	PI	4.0 mg
	PS	1.7 mg
	PA	3.2 mg
	SM	2.2 mg
	LPC	6.7 mg
Cholesterol		4.5 mg
Purine (guanine)		1.4 mg

## Abbreviations

PLDP	porcine liver decomposition product
MCI	mild cognitive impairment
LPA	lysophosphatidic acid
LPE	lysophosphatidylethanolamine
LPC	lysophosphatidylcholine
LPS	lipopolysaccharide
cPA	cyclic phosphatidic acid
ROS	reactive oxygen species
CNS	central nervous system
